# Multidisciplinary Integrative Medicine Approach for Cancer Patients: A Multicenter Retrospective Study

**DOI:** 10.3390/nu17061012

**Published:** 2025-03-13

**Authors:** Massimiliano Berretta, Vincenzo Quagliariello, Alessandro Ottaiano, Mariachiara Santorsola, Raffaele Di Francia, Patrizia Carroccio, Nicola Maurea, Oreste Claudio Buonomo, Gaetano Facchini, Giordana Di Mauro, Monica Montopoli, Enrica Toscano, Claudia Gelsomino, Antonio Picone, Tindara Franchina, Paola Muscolino, Alessia Bignucolo, Gianluca Vanni, Giuliana Ciappina, Liliana Montella

**Affiliations:** 1 Department of Clinical and Experimental Medicine, University of Messina, 98125 Messina, Italy; alessia.bignucolo@unime.it (A.B.); 2 Integrative Medicine Research Group (IMRG), Noceto, 43015 Parma, Italy; monica.montopoli@unipd.it (M.M.); 3 Division of Medical Oncology, Policlinico “G. Martino” Hospital, University of Messina, 98125 Messina, Italy; antonio.picone@unime.it (A.P.); tindara.franchina@unime.it (T.F.); giuliana.ciappina@polime.it (G.C.); 4 Division of Cardiology, Istituto Nazionale Tumori, Fondazione “G. Pascale”, 80131 Naples, Italy; vincenzo.quagliariello@istitutotumori.na.it (V.Q.); n.maurea@istitutotumori.na.it (N.M.); 5 SSD-Innovative Therapies for Abdominal Metastases, IRCCS “G. Pascale”, Istituto Nazionale Tumori di Napoli, Via M. Semmola, 80131 Naples, Italy; a.ottaiano@istitutotumori.na.it (A.O.); mariachiara.santorsola@istitutotumori.na.it (M.S.); 6 Gruppo Oncologico Ricercatori Italiani (GORI-Onlus), 33170 Pordenone, Italy; rdifrancia@iapharmagen.org (R.D.F.); 7 School of Specialization in Medical Oncology, Department of Human Pathology “G. Barresi”, University of Messina, 98125 Messina, Italy; patrizia.carroccio@studenti.unime.it (P.C.); giordana.di.mauro@studenti.unime.it (G.D.M.); enrica.toscano@studenti.unime.it (E.T.); claudia.gelsomino@studenti.unime.it (C.G.); paola.muscolino@studenti.unime.it (P.M.); 8 Breast Unit, Department of Surgical Science, PTV Policlinico Tor Vergata University, 00133 Rome, Italy; oreste.buonomo@ptvonline.it (O.C.B.); gianluca.vanni@ptvonline.it (G.V.); 9 Dipartimento di Scienze della Salute, University of Basilicata, Via Nazario Sauro, 85, 85100 Potenza, Italy; 10 Division of Medical Oncology, “Santa Maria delle Grazie” Hospital, ASL Napoli 2 Nord, 80078 Pozzuoli, Italy; gaetano.facchini@aslnapoli2nord.it (G.F.); liliana.montella@aslnapoli2nord.it (L.M.); 11 Department of Pharmaceutical and Pharmacological Sciences, University of Padova, 35131 Padova, Italy

**Keywords:** complementary integrative medicine, multidisciplinary approaches, cancer patients, quality of life, cancer-related fatigue, survival

## Abstract

Background: The use of complementary integrative medicine (CIM) by cancer patients is currently very common. The main reasons why patients turn to CIM are to improve quality of life (QoL) and support the immune system. Unfortunately, many patients rely on CIM self-prescription, neglecting the risk of interactions with anticancer treatments (ACTs). The primary objective is to demonstrate the feasibility of combining CIM and ACT in a multidisciplinary approach to improve the QoL of cancer patients and to reduce ACT’s adverse events. Methods: Cancer patients were treated with CIM by expert physicians. CIM mainly consisted of vitamins C and D, the medicinal mushrooms blend U-CARE, and probiotics administered alone or in combination. The patients were followed-up by physicians and data were recorded in a detailed shared file. Results: A total of 54 cancer patients were treated with an integrative approach, especially during ACTs. The combination showed a good safety profile. No adverse events occurred in 92.6% of patients, whereas only 7.4% of patients experienced gastrointestinal or liver toxicity from the CIM approach. The main benefit of the CIM approach was improved fatigue and QoL, and this was mainly achieved by the concomitant use of polytherapy-based complementary medicine (PCM) and U-CARE. The toxicity improvement was mainly associated with the use of solely U-CARE. Conclusions: These results highlight the feasibility of the CIM approach in cancer patients addressed by a multidisciplinary team of experts in the field. The patient-centered and evidence-based approach of CIM is an example of the comprehensive and coordinated strategy pursued by the EU in its programmatic document against cancer aiming to focus on the QoL of patients and to avoid potentially harmful CIM self-prescription.

## 1. Introduction

According to the National Center for Complementary and Integrative Health (NCCIH), the integrative medicine (IM) approach combines traditional medicine (TM) with complementary medicine (CM) approaches shown to be safe and effective [1]. Despite the concomitant use of complementary approaches with oncological treatments being controversial, a large proportion of cancer patients (20–50%) use them to manage the adverse events (AEs) of anticancer treatments (ACTs) or even to treat cancer [2,3,4,5]. Complementary and integrative medicine (CIM) approaches are highly heterogeneous, including a wide range of practices and biological products such as herbs/botanicals, vitamins, probiotics, and medicinal mushroom blends and non-biological products such as meditation and yoga (according to the five categories identified by the NCCIH) [1]. A major concern is patients’ sources of information about CIM: the main source is the media (48%), followed by friends and relatives (19%), and only 6% by healthcare practitioners [3,6]. Nevertheless, the physician remains the leading figure that patients trust and want to be guided by in their oncological decision-making. In contrast, only 48% of physicians are in favor of using the CIM approach, which is mainly due to an individual interest in this topic [7]. The limited knowledge of CIM also depends on the lack of university courses on the topic in many countries, which could enable a new generation of doctors to meet patients’ needs and avoid potentially harmful self-prescribing [7]. The self-prescription of CIM, indeed, may increase the risk of unexpected toxicities, and the failure of anticancer treatments (ACTs), due to pharmacokinetic or pharmacodynamic interactions between TM and CIM. The supervised use of evidence-based CIM approaches may improve quality of life (QoL), adherence to ACTs [5], and in some cases also overall survival (OS) in cancer patients, likely due to the reduction in ACT-related adverse events (AEs) causing therapy discontinuation or delay [2]. A European Union (EU) programmatic document updated in 2022 on cancer indicated that science-based CIM could be useful to improve QoL, but it was recommended that such approaches should be supervised by healthcare institutions and physicians [8]. Based on these considerations, we describe a multicenter retrospective study of multidisciplinary CIM approaches in 54 cancer patients based on good clinical practice and according to the guidelines for CIM approaches [9,10]. The main objective of our research was to demonstrate the feasibility of a CIM approach in cancer patients through a multidisciplinary work. To this end, the endpoints focused on improving patients’ QoL, reducing the side effects of ACTs, and ensuring the absence of AEs of complementary and integrative medicine. Secondly, in this—albeit small—patient group, we investigated how the improvement in managing ACTs potentially reflected on survival. The selection of the CIM approach depended on the clinical characteristics of the patients and the personal clinical experience of the physicians involved in the decision to use the CIM approach. As with ACTs, the CIM approach does not provide the same treatment for all cancer patients. Personalized CIM treatments based on parameters such as age, Eastern Cooperative Oncology Group performance status (ECOG PS), comorbidities, cancer-related symptoms, treatment failure, ACT cycles received, AEs, and terminal illness were the strategies adopted by the multidisciplinary team for the 54 patients. Each CIM approach was shared and discussed with the patients and their relatives. The therapeutic rationale for administering a CIM approach, that in our study mainly consisted of natural products, was to improve QoL, reduce toxicities, and support the immune system to counteract disease progression.

## 2. Materials and Methods

### 2.1. Patients and CIM Approach

This retrospective study included patients regardless of gender, age, cancer type, and stage who have required a multi-target supportive treatment (ST) from their physician since 2021. Three oncologists closely monitored the 54 patients, although the multidisciplinary team also included radiotherapists, pharmacists, and nutritional biologists who helped to tailor the CIM approach. Patients received standard ACTs before or during the CIM and were treated with a CIM approach after the failure of ACTs and/or the appearance of serious and/or persistent ACT-related AEs. The CIM mainly consisted of vitamin C (VitC) and D (VitD), probiotics, and a blend of medicinal mushrooms (MMs) (Micotherapy U-CARE, owned by ADV Reform srl, Noceto, Italy) administered alone or in combination. In a few isolated cases, glutathione was also given. VitC was orally administered with a maximum of 2 g daily, except for one patient for whom the i.v. administration was preferred (4 g i.v. daily). VitD was orally administered based on basal plasma vitamin D levels, and the doses ranged from 60,000 IU monthly to 100,000 IU monthly. Probiotics were prescribed according to specific patients’ needs. The most used strains were as follows: *Lactobacillus rhamnosus* LRH11, *Lactobacillus rhamnosus* GG, *Bifidobacterium lactis* BB12, *Lactobacillus acidophilus* LA5, *Lactobacillus acidophilus* LA14, *Enterococcus faecium* EF41, and *Saccharomyces cervisae subspecies boulardii*. Medicinal mushroom Micotherapy U-CARE has been registered by the Italian Ministry of Health as a dietary supplement (registration number 627 I.5.i.h.2/2020/627). It is produced from a hot water extract which is precipitated with ethanol and then freeze-dried. Each capsule contains a mixture of *Ganoderma lucidum, Grifola frondosa, Agaricus Blazei Murril, Cordyceps synensis,* and *Lentinula edodes* in equal amounts (300 mg) and titrated to a total of >30% of polysaccharides and >15% of β-(1,3)-D-glucans and β-(1-6)-D-glucans. The planned oral dose was 2 capsules daily between meals. Glutathione was only administered in cases of severe liver and kidney toxicity due to ACT or in some cases where ACT was suspended. Patients were treated in accordance with the Helsinki Declaration of 1964 and its subsequent amendments or equivalent ethical standards and local ethical guidelines. Patients were fully informed of the proposed CIM approach and signed the informed consent form. This study followed the reporting guidelines for studies [11].

### 2.2. Rationale for the CIM Approach Selection

Each CIM approach was closely patient-tailored, as stated in the Introduction Section of this manuscript. The CIM approach consisted of VitC and VitD, a medicinal mushrooms blend, probiotics, and glutathione. VitC improves HR-QoL and attenuates cancer-related adverse events. Despite most clinical benefits being provided by iv administration, the oral intake of VitC also enhanced HR-QoL [12]. In addition, VitC’s immunomodulatory activity may be a promising integrative therapy in combination with immunotherapy as at high doses, it enhances immune checkpoint agents and increases CD8 T cell infiltration and cytotoxicity in the tumor microenvironment [12]. The use of VitD is supported by observational studies which have demonstrated that it is associated with longer survival in cancer patients. Notably, VitD reduces the lipid peroxidation usually involved in cardiovascular and neurodegenerative diseases, as well as in cancer [13]. Recently, the role of VitD as a regulator of immune function has been elucidated, which is important in patients with a deregulated immune system such as cancer patients. In particular, VitD has been shown to be an important modulator of the expression of genes that regulate both the innate and adaptive immune systems [14]. It is known that the VitD receptor is expressed in several tumor cells, tissues, and, particularly, in the stroma of the tumor microenvironment, and we can therefore assume that it exerts a direct antitumor effect [13]. Particularly, its antitumoral effects include the inhibition of cell cycle, cell differentiation and apoptosis, autophagy, and angiogenesis via the regulation of signaling pathways such as PI3K/Akt/ERK1/2/MAPK, WNT/β-catenin, and nuclear factor kappa-light-chain-enhancer of activated B cells (NF-κB) signaling [15]. Due to both antitumor and anti-inflammatory activities in the tumor microenvironment, VitD represents a proper CIM approach to control tumor progression and enhance ACTs. Two recent metanalysis highlighted the negative impact of low VitD levels on time to outcome both in stage III CRC and metastatic CRC patients [16,17]. In addition, a metanalysis focused on BC patients emphasized that adequate pretreatment levels of VitD had a positive impact on progression-free survival (PFS) and response to neo-adjuvant chemotherapy [18]. Probiotics are non-pathogenic live microorganisms that play an active role in improving the health of the host when taken at the right amount [19]. Research on probiotics and their role in oncology highlights several promising aspects of their use in cancer prevention and treatment. Probiotics have shown potential to enhance anticancer immunity by modulating the gut microbiota and immune system [20]. Some studies emphasize the role played by probiotics in different cancer types, including CRC, oral, oropharyngeal, and skin cancers [21,22,23]. Probiotics modulate cancer through various pathways, including the downregulation of oncogene expression, inhibition of kinases, induction of autophagy, and reactivation of tumor suppressors [23]. By promoting healthy or immune-potentiating microbiota, probiotics help the immune system to detect and eliminate cancerous cells. Probiotics can improve the efficacy of conventional cancer treatments such as chemotherapy and immunotherapy while potentially reducing their AEs. They also contribute to cancer therapy by enhancing immune barrier function, inhibiting carcinogenic enzymes, promoting apoptosis, and maintaining redox homeostasis. Additionally, probiotics produce metabolites with anticarcinogenic properties that can act directly on cancer cells and indirectly as immunomodulatory signals [20]. The role of medicinal mushroom (MM) blends is more intriguing. Medicinal mushrooms have been used in TM for centuries, particularly in Asian cultures, and they are gaining popularity in modern clinical applications due to their potential health benefits. According to the NCI and the NCCIH, they can improve the HR-QoL, microbiota diversity, and immunomodulation [24,25]. Moreover, they were approved in addition to standard ACTs in Japan and China more than 30 years ago and have an extensive history of safe clinical use alone or combined with radiotherapy or ACTs [26,27]. The MM blends contain numerous bioactive compounds, including beta-glucans, triterpenes, ergothioneine, and ergosterols that influence several cancer-related pathways, often synergistically, by modulating the cellular targets involved in cell proliferation, survival, and angiogenesis [28]. For example, Reishi (*Ganoderma Lucidum*) and *Trametes Versicolor* (Turkey tail) exert hepatoprotective properties that can protect the liver from chemotherapy- and immunotherapy-related damages [29,30]. Medicinal mushrooms exert immune-enhancing activities via the stimulation of growth and differentiation of lymphocytes [31]. Trametes Versicolor extract, indeed, can increase CD8+ T cells and CD19+ B cells in BC patients. Reishi significantly increases IFN-γ, IL-2, IL-6, and NK cell (CD56+ cells) levels in lung cancer patients, thereby potentially increasing immune therapies [28]. Some mushrooms like Reishi, Cordyceps sinensys, Agaricus Blazei Murril, and Maitake reduce TH-2 cytokines with anti-inflammatory and immune-enhancing functions. Protein complexes, polysaccharides, and β-glucans of MM reduce NF-kB expression in antigen-presenting cells (APCs) and increase the synthesis and release of interferon-gamma (INF-γ) from natural killer (NK) cells and TH-1 pathways, which leads to the activation of macrophages and cytotoxic lymphocytes with antitumor properties [28]. Mushroom blend extracts, such as U-CARE, exert their immunomodulating activity via branched-β1,3-β1,6 β-glucans due to their tertiary triple-helix structure and high conformational heterogeneity [32]. The diversity of branched-β-glucans contained in the mushroom mixture is transferred to APCs such as macrophages, dendritic cells (DCs), and NK cells by enhancing their non-self-recognition capabilities through the stimulation of specific receptors located on the cell membrane [33]. β-glucans also have a high prebiotic effect, which is mediated by microbiota. Particularly, β-glucans are degraded by bifidobacteria via a complex of glucanases anchored to a transmembrane domain. The blend of β-glucans thus exerts bifidogenic activity, increasing the robustness and biodiversity of the microbiota and the production of short-chain fatty acids (SCFAs), improving mucosal integrity, nutrient absorption, and drug response [34,35]. The incorporation of MMs into clinical practice is increasing, reflecting the merging of traditional knowledge and modern scientific research. Glutathione, a tripeptide widely present in tissues and cells, is preferentially concentrated in the liver, where its biologically active reduced form (GSH) plays an antioxidant and detoxifying role for cell homeostasis [36]. Given the antioxidant effect of GSH, its role in cancer therapy is twofold: (a) as a detoxification agent by conjugating xenobiotics and (b) the activation of some prodrugs in active metabolites [37]. However, in some cases, its role is controversial, like for the association of doxorubicin (DOX) and glutathione. Whilst the administration of exogenous GSH during DOX-based chemotherapy protects from hepatotoxicity and cardiotoxicity, on the other hand, it significantly decreases the antineoplastic effectiveness of DOX in a dose-dependent manner, thus proving the disadvantageous effect of GSH use [38]. In general, the administration of glutathione or its precursor N-acetylcysteine (NAC) is widely used to reduce the AEs of chemotherapy. The use of glutathione in our study was evaluated for each individual patient based on the type of chemotherapy and potential pharmacokinetic and pharmacodynamic effects with the drugs.

### 2.3. Data Collection

Patients’ data were collected in a shared file, continuously updated from the three oncologists who followed-up the patients. Only those prescribing CIM were allowed to access the shared file. The data collected included patients’ biographical and clinical data, type of ACT, PS at diagnosis and at the start of the CIM treatment, type of CIM treatment, goals of the CIM treatment and benefits achieved, and any AEs. In particular, among AEs, we evaluated cancer-related fatigue (CRF) in patients integrating supplements, employing a validated assessment tool widely used in oncology research called the Brief Fatigue Inventory (BFI). In brief, the BFI was administered to assess the severity and impact of fatigue on daily activities. This self-reported questionnaire consists of nine items, including the worst fatigue in the last 24 h and its interference with mood, walking ability, normal work, and enjoyment of life. Fatigue assessments were conducted at the baseline (before supplement initiation) and at scheduled follow-up visits to monitor changes over time.

### 2.4. Statistical Analysis

All the data were reported into the prospective Excel database (Microsoft, Washington, DC, USA version 16.78, 2023). Based on the presence or absence of locoregional recurrence, clinicopathological variables were compared between the groups using the T test for continuous variables. Fisher’s exact test was applied in cases of dichotomous variables, and the Monte Carlo test was used in cases of non-dichotomous variables. Multivariate logistic regression analysis was performed to identify the risk predictors of DCIS recurrence. Only *p*-value < 0.05 was statistically significant. The Kaplan–Meier curve was adopted to evaluate recurrence, and the log-rank test was used to assess statistically significant differences between the groups. A logistic regression statistical model was used to estimate the effect of factors on LLR. Pearson’s test was adopted to appraise the correlation between the variables. All the statistical analyses were performed in SPSS statistical package version 23.0 (SPSS Inc., Chicago, IL, USA).

## 3. Results

### 3.1. Clinicopathological Profile and Treatment Landscape of Oncology Patients Receiving Complementary Medicine

The heterogeneity of the patient population in terms of clinical characteristics, disease stage, treatment modalities, and metastatic patterns, highlighting the complexity of managing oncological patients who explore CIM approaches, is detailed in Table 1. The analysis included 54 patients, with a median age of 62 years, ranging from 28 to 83 years. Most patients were younger than 70 years (75.9%). Females made up a slight majority, representing 57.4% of the cohort. The most common types of cancer were BC (25.9%), CNS tumors (20.4%), and CRC (16.6%). Other cancers included urogynecological malignancies (11.1%), non-colorectal gastrointestinal tumors (7.4%), hematologic malignancies (7.4%), and a small percentage of lung, multiple cancers, or less common cancers (3.7%). Advanced-stage disease was predominant, seen in 83.3% of cases, while early-stage cancers accounted for 16.7%. Histological grading revealed that 68.5% of tumors were high-grade (G2/3), while 31.5% were low-grade (G1). Metastatic disease was common, with 33.3% of patients presenting multiple metastatic sites. Uncommon metastatic locations, such as cutis, adrenal glands, spleen, and ocular tissues, were observed in 14.8% of cases. Notably, 20.3% of patients had no metastases at the time of analysis. The ECOG PS was favorable for most patients, with 77.7% having a score of 0–1, indicating good functional capacity. A diverse range of ACTs was administered, with chemotherapy being the most common (37.7%). Hormone therapy (14.8%), targeted therapies (9.2%), and anti-angiogenic drugs (7.4%) were also employed. Only one patient received immunotherapy (1.8%) or radiotherapy (1.8%). Combinational treatment regimens were administered to 24.1% of the cohort, while 3.7% did not receive any active treatment.

The distribution of lifestyle factors and comorbidities among the study population may highlight distinct patterns relevant to CIM in oncology (Table 2). Interestingly, nearly half of the patients (48.1%) adhered to a healthy lifestyle, which was the most prevalent category. Sedentary behavior was observed in 31.5% of the population, indicating a significant proportion with limited physical activity. A smaller segment, comprising 13.0%, reported an active lifestyle. Smoking was reported by only 7.4% of patients, reflecting the low prevalence of this risk behavior in the cohort. In terms of comorbidities, most participants (61.1%) had no concurrent medical conditions, suggesting a relatively healthy baseline in a significant portion of the population. Among those with comorbidities, cardiovascular conditions were the most frequently reported (14.8%), followed by metabolic disorders (9.3%). Liver conditions and anxiety–depression syndrome were equally represented at 5.6% each. A minority of participants (3.7%) presented with multiple comorbidities, reflecting a more complex health status.

### 3.2. Oncological Treatment Duration, Toxicity, and Responses in Patients Receiving CIM

The results presented in Table 3 provide a comprehensive overview of the duration of ACTs, the spectrum of treatment-related AEs, and therapeutic responses in patients who received CIM alongside standard ACTs. These data underscore the potential for CIM to coexist with ACTs, providing insights into treatment duration, tolerability, and therapeutic efficacy. Regarding treatment duration, most patients (59.2%) underwent ACTs for over 12 months, suggesting sustained therapy for many patients. Conversely, approximately 20.4% of patients each reported treatment durations of less than 6 months or between 6 and 12 months, indicating variability in treatment continuity. Regarding ACTs toxicity, most patients experienced manageable AEs, with 64.8% reporting toxicity levels below grade 2 (G2). Severe toxicities (above grade 3) were rare, occurring in only 3.7% of patients. Interestingly, 31.5% of patients did not report any toxic events, highlighting the tolerability of the treatment regimens in a significant subset of the cohort. Notably, 20.4% of patients achieved a CR, and 38.7% showed a PR, collectively accounting for nearly 60% of patients who experienced meaningful tumor reduction. This positive result is obviously influenced by the presence of gastrointestinal, urogynecological, and hematological neoplasms. Stable disease was observed in 34.7% of patients, indicating disease control in these cases, while 6.1% of patients experienced disease progression.

### 3.3. Implementation of CIM Approach in the Oncological Practice

Table 4 describes several qualitative and quantitative relationships between ACTs and CIM. At the initiation of CIM, most patients (72.2%) had an ECOG PS of 0–1, indicating relatively good functional status, while a smaller subset (25.9%) had more impaired performance with ECOG PS 2–3. The type of CIM varied, with nearly half of the patients (46.3%) utilizing a combination of U-CARE and polytherapy-based complementary medicine (PCM), followed by 40.7% opting solely for U-CARE and 12.9% relying exclusively on polytherapy-based CIM. CIM approaches were predominantly associated with systemic ACTs, with 37% of patients starting during the first line of cancer therapy, 31.4% in the second line, and 22.2% beyond the second line. Only a minority of patients (5.6%) used CIM in an adjuvant setting, and 3.8% were not undergoing systemic therapy. In terms of timing, CIM was most frequently initiated during ACTs (72.2%), while smaller proportions began at the time of diagnosis (7.4%), at the initiation of ACTs (11.1%), or following its completion (9.3%). Patients reported different motivations for starting CIM. General support was the most common reason (40.7%), followed by support for managing AEs (12.1%) and immunomodulation (1.8%). Notably, nearly half (44.4%) pursued CIM for a combination of these reasons. The duration of CIM administration was evenly distributed, with 37% receiving treatment for less than 3 months, 25.9% for 3–6 months, and another 37% for more than 6 months, reflecting a mix of short- and longer-term engagement with these therapies.

### 3.4. Clinical Benefit, Toxicity, and Costs of Cim Approach

The analysis of clinical outcomes, toxicity, and costs associated with CIM administration is reported in Table 5. Regarding clinical benefits, most patients (44.4%) experienced an improvement in fatigue and overall QoL. Improvements in either QoL or ACT-related toxicity were reported in 11.1% of patients for each category, while 11.1% of patients reported no benefit from the CIM therapies. Smaller subsets of patients experienced combined benefits, including fatigue and toxicity relief (3.7%), tumor shrinkage alone (1.8%), or in combination with QoL improvement (1.8%). A subset of 12.1% reported multiple benefits spanning several categories, highlighting the potential multidimensional impact of these treatments in certain cases. Toxicity associated with CIM was minimal. Nearly ninety-three percent (92.6%) of patients reported no AEs. Among those who experienced toxicity, the most common AEs were gastrointestinal (5.5%) and liver-related toxicity (1.8%). Notably, pharmacological interaction testing, performed in 17 patients, did not include any of those who experienced toxicity, underscoring a potential area for further investigation. Cost analysis showed that CIM was generally affordable, with 74.1% of patients incurring monthly expenses of less than EUR 100. However, 35.9% of patients reported costs exceeding EUR 100 per month, indicating variability in financial burden depending on the type of IM prescribed.

### 3.5. Factors Influencing Response to Cim and Survival

**Figure 1:**
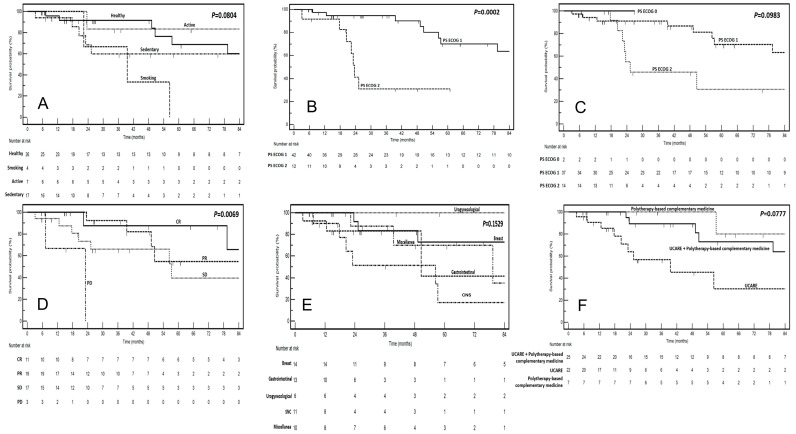
Kaplan–Meier curves illustrating survival stratified by the following factors are presented: (**A**) lifestyle, (**B**) PS ECOG at cancer diagnosis, (**C**) PS ECOG at the start of complementary therapy, (**D**) best response, (**E**) cancer type, and (**F**) type of complementary therapy. Log-rank test *p*-values are reported in the corresponding figures. The number of events is indicated at the bottom of each figure.

The univariate analysis examined factors associated with clinical benefit from CIM and their potential impact on OS (Table 6). Among the variables analyzed (age, gender, lifestyle, type of cancer, type of CIM and its duration, ECOG PS at cancer diagnosis or at CIM initiation, cancer stage, timing of CIM initiation, setting of therapy, and best response to ACT), none were significantly associated with clinical benefit from CIM. The Kaplan–Meier survival curves stratified by these factors are reported in Figure 1. However, ECOG PS at cancer diagnosis and best response to oncological treatment were significantly associated with survival outcomes. Specifically, patients with an ECOG score of 2 had a markedly higher risk of death (HR: 16.8, 95% CI: 3.78–74.5, *p* = 0.0002) compared to those with a PS ECOG of 1. Additionally, patients with progressive disease had significantly worse survival outcomes compared to those achieving a complete response (HR: 11.91, 95% CI: 0.29–48.37, *p* = 0.0076).

### 3.6. Associations Between Response to Act, Cancer Type, and Cim

The analyses included in Table 7 were conducted for descriptive purposes only. Regarding cancer types, patients with BC and gastrointestinal cancers showed more favorable responses to oncological treatments, with higher frequencies of complete response (CR) and partial response (PR) compared to other cancer types. In contrast, patients with CNS cancers predominantly exhibited stable disease (SD) or progressive disease (PD), with no CR or PR observed. Similarly, patients with urogynecological cancers had limited favorable responses, while the “miscellanea” group (including hematologic malignancies) demonstrated the most notable distribution of CR and PR outcomes, reflecting heterogeneity within this category (*p* = 0.0019). Regarding the CIM approach, the combination of U-CARE and PCM was associated with a higher proportion of favorable responses, including 6 cases of CR and 11 cases of PR, compared to either U-CARE or PCM alone. Specifically, U-CARE alone resulted in fewer CRs and PRs and a higher prevalence of SD, while PCM alone also produced fewer CRs and PRs but showed no cases of PD. The differences among these groups were statistically significant (*p* = 0.041).

### 3.7. Factors Predicting OS in Cancer Patients Treated with CIM

A multivariate analysis was performed following a careful and consensus-based selection of specific variables (Table 8). Among the variables evaluated, PS, as assessed by ECOG at the time of cancer diagnosis, emerged as a statistically significant predictor of survival outcomes. Patients with a PS of 2 exhibited a hazard ratio (HR) of 4.86 (95% CI: 1.24–19.0, *p* = 0.0232), indicating a more than fourfold increased risk of mortality compared to those with a PS of 0–1. This finding highlights the critical role played by initial functional status in determining survival outcomes.

### 3.8. Comparative Efficacy of CIM in Oncology: Cancer-Specific Applications and Multidimensional Clinical Benefits

The results summarized in Table 9 highlight significant differences in the distribution of cancer types and the clinical benefits associated with CIM, specifically PCM, U-CARE, and their combination (U-CARE + PCM). These therapies exhibit distinct patterns of utilization and effectiveness across cancer types and clinical outcomes. BC was the most frequently treated with U-CARE + PCM, with eight cases, compared to three cases for PCM and U-CARE individually, suggesting either a preference for or enhanced efficacy of the combined approach for this cancer type. In contrast, CNS cancers predominantly relied on U-CARE alone, with 10 cases, while PCM or U-CARE + PCM use was minimal or absent. Gastrointestinal cancers exhibited a more balanced distribution, with moderate use across all three therapy modalities. Notably, urogynecological cancers were exclusively treated with U-CARE + PCM, indicating a potential unique suitability of this combination for such malignancies. For miscellaneous cancers, the distribution was more varied, although the combined approach remained prominent. These variations in therapy applications across cancer types were statistically significant (*p* = 0.004). The distribution of clinical benefits further highlights the distinct contributions of each therapy. Improvements in fatigue and QoL were overwhelmingly associated with U-CARE + PCM, with 20 cases compared to 3 for PCM and 1 for U-CARE. Conversely, improvements in toxicity were most notable with U-CARE alone (four cases), whereas PCM showed no efficacy in this area. The combined U-CARE + PCM approach demonstrated the broadest efficacy, providing benefits across multiple clinical categories.

## 4. Discussion

Scientific advances in oncology have led to the development of innovative drugs that can improve survival. The result is ACTs that can last for years and, while keeping the disease under control, can sometimes affect the patient’s QoL [39]. Patients themselves often turn to CIM to reduce ACT-related side effects and improve QoL, often relying on non-evidence-based sources with the risk of compromising cancer therapies and incurring unwanted toxicities [3,40]. Today, there is a strong need to improve QoL in cancer patients, especially cancer survivors. For this reason, it is necessary for healthcare professionals, especially oncologists, to increase their knowledge of CIM and begin to feel comfortable with these complementary therapies [7]. This effort is required not only by patients themselves, who see the oncologist as a key reference figure in their treatment decision-making, but also by the European Union, which in a policy document in 2022 emphasized the importance of shared recommendations on integrated supportive care, including evidence-based complementary therapies [8]. A relevant aspect is the need to develop a patient-centered and -informed CIM approach in which the use of complementary therapies is decided by an experienced multidisciplinary team and under the ongoing supervision of healthcare professionals. To this end, it is necessary for health professionals to be continuously updated in the field of integrated medicine, perhaps even by introducing specific courses at university level to train future professionals capable of meeting the needs of cancer patients.

From a biochemical point of view, mycotherapy and probiotics exert beneficial biochemical effects in cancer patients by modulating immune responses, reducing inflammation, enhancing gut microbiota composition, and influencing the metabolic pathways associated with tumor progression. Medicinal mushrooms contain bioactive compounds such as beta-glucans, triterpenes, and polysaccharides that interact with pattern recognition receptors (PRRs) like Dectin-1 and Toll-like receptors (TLRs) on macrophages and dendritic cells [41]. This interaction stimulates NF-κB and MAPK signaling pathways, leading to the increased production of pro-inflammatory cytokines such as TNF-α, IL-6, and IL-12, which enhance cytotoxic T cell activity while reducing immunosuppressive factors like IL-10. Additionally, beta-glucans activate the complement system via the alternative pathway, enhancing phagocytosis and natural killer (NK) cell-mediated cytotoxicity against tumor cells [42]. Mycotherapy also modulates oxidative stress through the activation of the Nrf2-ARE pathway, which upregulates antioxidant enzymes such as superoxide dismutase (SOD) and glutathione peroxidase (GPx), counteracting reactive oxygen species (ROS)-induced DNA damage that contributes to cancer progression. Polysaccharides and triterpenes in medicinal mushrooms, such as ganoderic acids from *Ganoderma lucidum*, inhibit angiogenesis by downregulating VEGF and HIF-1α expression, thereby limiting tumor vascularization and nutrient supply [43]. Certain fungal-derived metabolites also exhibit direct apoptotic effects via mitochondrial cytochrome c release and caspase activation, modulating intrinsic apoptotic pathways in malignant cells [44]. Probiotics influence cancer progression through microbiota–host interactions that regulate immune function and metabolic homeostasis. *Lactobacillus* and *Bifidobacterium* species produce short-chain fatty acids (SCFAs), particularly butyrate, which serve as histone deacetylase (HDAC) inhibitors, promoting the epigenetic modulation of genes involved in apoptosis and cell cycle arrest [45]. Butyrate also enhances regulatory T cell (Treg) differentiation via the activation of G-protein-coupled receptors (GPR41/GPR43), contributing to immune homeostasis. Moreover, probiotics enhance intestinal barrier integrity by upregulating tight junction proteins such as occludin and claudin, preventing bacterial translocation and systemic inflammation. Moreover, probiotic-derived metabolites such as indole derivatives and polyamines modulate inflammatory signaling by inhibiting NF-κB and reducing the production of pro-inflammatory cytokines like IL-6 and IL-1β, which are often elevated in cancer-associated inflammation [46]. Some bacterial strains, such as *Lactobacillus rhamnosus*, could promote the expression of mucins (MUC2, MUC5AC), which create a protective mucus layer, reducing the risk of pathogen-induced oncogenic inflammation. The gut microbiota also influences tryptophan metabolism, shifting kynurenine pathway flux toward the production of immunosuppressive metabolites that affect T cell function in the tumor microenvironment [47]. Therefore, by integrating mycotherapy and probiotics, cancer patients could experience synergistic benefits that enhance immune surveillance, mitigate oxidative stress, and reduce chronic inflammation, ultimately contributing to improved treatment outcomes and QoL [48].

This retrospective multicenter study described the implementation of a CIM approach in the oncology field carried out by a multidisciplinary team. The ability to manage the prescription of CIM by a specialized team, working in close collaboration, brought benefits to the majority of 54 patients, in terms of improved QoL, cancer-related fatigue, and adherence to ACTs, while limiting harmful self-prescription [49]. Our study showed a safety profile for the combination of CIM approaches and ACTs. Only 5.6% of patients reported gastrointestinal toxicity and 1.8% hepatic toxicity, highlighting a reasonable safety profile. Additionally, in 17 out of 54 patients, regardless of the appearance of toxicity, drug–drug interaction tests were performed using online checker tools to ensure no interference with the ACTs [50,51]. As shown by the results, a considerable proportion of patients (44.4%) who received CIM reported a reduction in cancer-related fatigue and an improvement in QoL, which may have contributed to better adherence and continuity with ACTs. While the improved management of ACT side effects and fatigue is expected to support treatment adherence by reducing delays or discontinuation, any potential association with survival outcomes should be interpreted with caution, given this study’s sample size. The results of this retrospective study demonstrate that the combination of U-CARE and PCM was associated with a higher proportion of favorable responses, including 6 cases of CR and 11 cases of PR, compared to either U-CARE or PCM alone. Although the literature data regarding the benefits in terms of survival outcomes with the use of CIM in early stages of disease are conflicting [52], in our study, with the limitations of the small number of patients in this setting (5.6%), no statistically significant differences were observed. Our study included only some of the practices encompassed by CIM, which may therefore lead to a lower incidence of adverse events compared to the data available in the literature [53]. Furthermore, most patients included in this study had a healthy lifestyle at the baseline (48.1%) and did not have comorbidities (61.1%), which reduced the risk of interactions even with non-oncological concomitant medications. The collected data provide a range of useful information, even in clinical practice, for the management of oncological patients before, during, and after ACTs. Moreover, the increasing use of CIM further emphasizes the importance of conducting targeted prospective studies aimed at gaining a more detailed understanding of the potential synergism between CIM and ACTs, as well as their long-term effects. In addition to conventional medical treatments, many patients, particularly those in non-curative settings, incorporate CIM as part of their therapeutic approach, aiming to improve overall survival and QoL. This phenomenon is well documented in oncology, where patients frequently use complementary interventions such as herbal medicine, acupuncture, mind–body therapies, and dietary supplements alongside standard treatments. Emerging evidence also suggests that cim plays a role in improving general survival and health outcomes in non-cancer populations. For instance, cardiovascular patients benefit from lifestyle-based complementary approaches, including meditation, yoga, and plant-based diets, which have been associated with improved cardiovascular function and reduced mortality risk. Similarly, in chronic illnesses such as neurodegenerative disorders and autoimmune diseases, CIM, including dietary interventions, probiotics, and antioxidant supplementation, has shown potential in mitigating disease progression and enhancing survival. While these approaches are widely adopted, their efficacy and safety require rigorous scientific validation through high-quality clinical trials. Integrating CIM into evidence-based practice may provide a holistic approach to patient care, emphasizing both disease management and overall longevity. There are some limitations to this study, among which some are based on its retrospective nature. Moreover, the included patients presented heterogeneous cancer types and different disease stages, which can partly affect the results. Selected CIM approaches also vary between patients, although this may be due to the personalization of the integrative treatments delivered in a patient-centered way. In many cases, the CIM approach consisted of the combination of multiple complementary treatments, whereas some patients were administered a single complementary treatment. Another limitation includes QoL and cancer-related fatigue evaluation being affected by the retrospective nature of this study. This parameter was collected based on the clinical assessment during the patients’ follow-up and the patients’ self-perceived health status without using a dedicated questionnaire.

## 5. Conclusions

This retrospective study suggests that the CIM approach may be a feasible strategy to improve the QoL of cancer patients as well as reduce toxicities and fatigue during or after ACTs regardless of both the type of cancer and the disease stage. A key element that emerged from this study was the multidisciplinary assessment carried out for each patient to select the most appropriate CIM treatment, and the presence of health professionals trained in CIM. The feasibility of a CIM approach to cancer care and the QoL of chronic and non-chronic cancer patients are the focuses of this study, but we also considered EU recommendations that emphasize the importance of developing an evidence-based, integrative, and patient-centered approach and, where appropriate, promoting the CIM under the supervision of health professionals [8]. Furthermore, to our knowledge, this is the first manuscript to describe a CIM strategy based primarily on a micotherapy approach by a multidisciplinary team of physicians who are experts in this field. Further well-designed and prospective studies are needed to confirm the benefits seen in this retrospective study and to better understand the mechanisms underlying the synergism between ACTs and CIM.

## Figures and Tables

**Table 1:** Clinicopathological characteristics of analyzed patients.

Variable	No. (%)
Age	
Median: 62 y; range (28–83 y)	
<70 y	41 (75.9)
≥70 y	13 (24.1)
Gender	
Female	31 (57.4)
Male	23 (42.6)
Type of cancer	
Breast	14 (25.9)
CNS	11 (20.4)
Colorectal	9 (16.6)
Urogynecological	6 (11.1)
GI non colorectal	4 (7.4)
Hematologic	4 (7.4)
Multiple cancers	2 (3.7)
Lung	2 (3.7)
Other	2 (3.7)
Stage	
Advanced	45 (83.3)
Early	9 (16.7)
Grading	
G1	17 (31.5)
G2/3	37 (68.5)
Metastatic sites	
Not applicable	11 (20.3)
Lymph nodes	6 (11.1)
Liver	4 (7.4)
Bone	3 (5.5)
Peritoneum/Pleura	3 (5.5)
Lung	1 (1.8)
Multiple sites	18 (33.3)
Other *	8 (14.8)
PS ECOG	
0–1	42 (77.7)
2–3	12 (22.2)
Type of oncological treatment	
Chemotherapy	20 (37.7)
Hormone therapy	8 (14.8)
Target therapy	5 (9.2)
Anti-angiogenic drugs	4 (7.4)
Immunotherapy	1 (1.8)
Radiotherapy	1 (1.8)
None	2 (3.7)
Combinations	13 (24.1)

* Other refers to uncommon metastatic sites, including cutis, adrenal glands, spleen, and ocular sites. CNS: central nervous system; ECOG: Eastern Cooperative Oncology Group; PS: performance status.

**Table 2:** Distribution of lifestyle factors and comorbidities in the study population.

Variable	No. (%)
Lifestyle	
Healthy	26 (48.1)
Sedentary	17 (31.5)
Active	7 (13.0)
Smoking	4 (7.4)
Comorbidities	
None	33 (61.1)
Cardiovascular	8 (14.8)
Metabolic	5 (9.3)
Liver	3 (5.6)
Anxiety–depression syndrome	3 (5.6)
Multiple morbidities	2 (3.7)

**Table 3:** Duration, toxicities, and responses to oncological treatments.

Variable	No. (%)
Oncological treatment duration	
<6 months	11 (20.4)
≥6–<12 months	11 (20.4)
≥12 months	32 (59.2)
Toxicity *	
≤G2	35 (64.8)
Hematologic	15
Non-hematologic	41
Fatigue	20
Gastrointestinal	13
Neurologic	3
Cutis	1
Renal	1
≥G3	2 (3.7)
Renal	1
Gastrointestinal	1
No toxicity	17 (31.5)
Best response **	
Complete response	10 (20.4)
Partial response	19 (38.7)
Stable disease	17 (34.7)
Progressive disease	3 (6.1)

* The total number of patients experiencing toxic events does not equal 35 because a single patient may experience more than one type of toxicity. ** The total number of patients does not amount to 54 because 5 patients received integrative treatment as part of an adjuvant therapeutic program (systemic or local).

**Table 4:** Oncological treatments and integrative medicine.

Variable	No. (%)
PS ECOG at the start of CIM	
0–1	39 (72.2)
2–3	14 (25.9)
Type of complementary therapy	
U-CARE + Polytherapy-based complementary medicine	25 (46.3)
U-CARE	22 (40.7)
Polytherapy-based complementary medicine	7 (12.9)
Line of therapy associated with CIM	
First	20 (37.0)
Second	17 (31.4)
Beyond second line	12 (22.2)
Adjuvant	3 (5.6)
No systemic therapy	2 (3.8)
Relationship between the timing of CIM and ACTs	
Initiation at the time of cancer diagnosis	4 (7.4)
Initiation at the onset of ACT	6 (11.1)
Initiation during ACT	39 (72.2)
Initiation following the completion of ACT	5 (9.3)
Motivations for initiating CIM	
General support	22 (40.7)
Support for toxicity	7 (12.1)
Immunomodulation	1 (1.8)
More than one of the above reasons	24 (44.4)
Duration of CIM	
≤3 months	20 (37.0)
>3–≤6 months	14 (25.9)
>6 months	20 (37.0)

ECOG: Eastern Cooperative Oncology Group; PS: performance status; U-CARE: Micotherapy U-CARE.

**Table 5:** Clinical benefit, toxicity, and costs of complementary products.

Variable	No. (%)
Type of clinical benefit	
Fatigue and QoL improvement	24 (44.4)
Toxicity improvement	6 (11.1)
QoL improvement	6 (11.1)
No benefit	6 (11.1)
Fatigue and toxicity improvement	2 (3.7)
Improvement in tumor shrinkage	1 (1.8)
Toxicity and QoL improvement	1 (1.8)
Improvement in tumor shrinkage and QoL	1 (1.8)
Multiple benefits	7 (12.1)
Type of toxicity *	
No toxicity	50 (92.6)
Gastrointestinal	3 (5.5)
Liver	1 (1.8)
Costs	
<EUR 100 per month	40 (74.1)
>EUR 100 per month	14 (35.9)

* A pharmacological interaction test was conducted in 17 patients, but it was not performed for any of the patients who experienced toxicity from CIM.

**Table 6:** Factors influencing response to CIM and survival (at univariate analysis).

Variable	Clinical Benefit from CIM		*p* (at Chi-Square test)	HR for OS	95% CIs	*p* (at Log-Rank Test)
	Yes	No				
Age						
<70 y	35	6		1		
≥70 y	13	0	0.1472	0.97	0.31–3.01	0.9663
Gender						
Male	20	3		1		
Female	28	3	0.6998	0.50	0.17–1.42	0.1960
Lifestyle						
Healthy	23	3		1		
Sedentary	16	1		1.72	0.57–5.14	
Active	6	1		0.91	0.24–3.33	
Smoking	3	1	0.7225	4.55	0.51–40.3	0.0804
Type of cancer						
Breast	13	1		1		
CNS	10	1		3.74	1.03–13.56	
Gastrointestinal	12	1		2.45	0.63–9.51	
Urogynecological	6	0		0.81	0.18–3.63	
Miscellanea	7	3	0.3101	1.73	0.46–6.49	0.1529
Type of complementary therapy						
U-CARE	23	2		1		
U-CARE + Polytherapy-based complementary medicine	18	4		0.49	0.18–1.36	
Polytherapy-based complementary medicine	7	0	0.3273	0.19	0.05–0.78	0.0777
Duration of CIM						
<3 months	17	3		1		
>3–≤6 months	13	1		0.75	0.24–2.34	
>6 months	18	2	0.7579	0.29	0.09–0.92	0.1551
PS ECOG at cancer diagnosis						
0	-	-				
1	37	5		1		
2	11	1	0.7309	16.8	3.78–74.5	0.0002
3	-	-				
PS ECOG at CIM start						
0	1	1				
1	32	5		1		
2	14	0	0.0844	2.46	0.83–7.28	0.0983
3	-	-				
Stage						
Early	8	1		1		
Advanced	40	5	1.0000	1.73	0.52–5.76	0.3684
Timing of CIM initiation						
At the time of cancer diagnosis	4	0		1		
At the onset of ACT	6	0		0.33	0.04–2.45	
During ACT	39	6		0.16	0.01–1.74	
Following the completion of ACT	5	0	0.4582	2.02	0.47–8.06	0.1831
Line of therapy associated with CIM						
First	18	2		1		
Second	14	3		1.23	0.37–4.12	
Beyond second line	11	1		1.37	0.39–4.76	
Adjuvant	2	0		1.19	0.29–4.81	
No systemic therapy	3	0	0.8303	1.26	0.14–11.0	0.9896
Best response to oncological treatment						
Complete response	9	1		1		
Partial response	17	2		1.43	0.44–4.57	
Stable disease	15	2		2.18	0.71–6.70	
Progressive disease	2	1	0.7186	11.91	0.29–48.37	0.0076

HR: hazard ratio; OS: overall survival; CNS: central nervous system; ECOG: Eastern Cooperative Oncology Group; PS: performance status; ACT: anticancer treatment.

**Table 7:** Associations between response to oncological treatments and type of cancer and CIM.

Variable	Best Response to Oncological Treatments				
	CR	PR	SD	PD	*p*
Type of cancer					
Breast	3	7	3	0	
CNS	0	0	8	2	
Gastrointestinal	2	7	2	0	
Urogynecological	0	1	3	1	
Miscellanea	5	4	1	0	0.0019
Type of complementary therapy					
U-CARE + Polytherapy-based complementary medicine	6	11	4	1	
U-CARE	1	6	12	2	
Polytherapy-based complementary medicine	3	2	1	0	0.041

CR: complete response; PR: partial response; SD: stable disease; PD: progressive disease; CNS: central nervous system; U-CARE: Micotherapy U-CARE.

**Table 8:** Multivariate analysis of selected prognostic factors influencing overall survival (OS) in cancer patients receiving CIM approach.

Variable	HR for OS	95% CIs	*p*
Age			
<70 y	1		
≥70 y	1.16	0.33–4.12	0.7236
Gender			
Male	1		
Female	0.46	0.15–1.38	0.2151
PS ECOG *			
0–1	1		
2	4.86	1.24–19.0	0.0232
PS ECOG **			
0–1	1		
2	3.15	1.18–8.36	0.3001
Type of cancer			
Breast	1		
CNS	3.76	1.08–14.6	0.0816
Gastrointestinal	2.46	0.63–10.06	0.1222
Urogynecological	0.81	0.17–3.84	0.2948
Miscellanea	1.83	0.44–6.52	0.3308
Type of complementary therapy			
U-CARE	1		
U-CARE + polytherapy-based complementary medicine	0.48	0.17–1.35	0.9117
Polytherapy-based complementary medicine	0.18	0.04–0.77	0.9882

CI: confidence interval; CNS: central nervous system; ECOG: Eastern Cooperative Oncology Group; HR: hazard ratio; OS: overall survival; PS: performance status; U-CARE: Micotherapy U-CARE. * at cancer diagnosis; ** at IM start.

**Table 9:** Distribution of cancer types and clinical benefits across complementary therapies.

Variable	Type of Complementary Therapy			
	PCM	U-CARE	U-CARE + PCM	*p*
Type of cancer				
Breast	3	3	8	
CNS	1	10	0	
Gastrointestinal	2	6	5	
Urogynecological	0	0	6	
Miscellanea	1	3	6	0.0041
Type of clinical benefit				
Fatigue and QoL improvement	3	1	20	
Toxicity improvement	0	4	2	
QoL improvement	1	5	0	
No benefit	1	5	0	
Fatigue and toxicity improvement	0	0	2	
Improvement in tumor shrinkage	0	1	0	
Toxicity and QoL improvement	0	1	0	
Improvement in tumor shrinkage and QoL	1	0	0	
Multiple benefits	1	5	1	0.0003

PCM: polytherapy-based complementary medicine, including a combination of probiotics, vitamins, curcumin, and aloe vera; QoL: quality of life; U-CARE: Micotherapy U-CARE; CNS: central nervous system.

## Data Availability

The original contributions presented in this study are included in the article. Further inquiries can be directed to the corresponding author.
